# Degradable transportation network with the addition of electric vehicles: Network equilibrium analysis

**DOI:** 10.1371/journal.pone.0184693

**Published:** 2017-09-08

**Authors:** Rui Zhang, Enjian Yao, Yang Yang

**Affiliations:** MOE Key Laboratory for Urban Transportation Complex Systems Theory and Technology, School of Traffic and Transportation, Beijing Jiaotong University, Beijing, China; Beihang University, CHINA

## Abstract

Introducing electric vehicles (EVs) into urban transportation network brings higher requirement on travel time reliability and charging reliability. Specifically, it is believed that travel time reliability is a key factor influencing travelers’ route choice. Meanwhile, due to the limited cruising range, EV drivers need to better learn about the required energy for the whole trip to make decisions about whether charging or not and where to charge (i.e., charging reliability). Since EV energy consumption is highly related to travel speed, network uncertainty affects travel time and charging demand estimation significantly. Considering the network uncertainty resulted from link degradation, which influences the distribution of travel demand on transportation network and the energy demand on power network, this paper aims to develop a reliability-based network equilibrium framework for accommodating degradable road conditions with the addition of EVs. First, based on the link travel time distribution, the mean and variance of route travel time and monetary expenses related to energy consumption are deduced, respectively. And the charging time distribution of EVs with charging demand is also estimated. Then, a nested structure is considered to deal with the difference of route choice behavior derived by the different uncertainty degrees between the routes with and without degradable links. Given the expected generalized travel cost and a psychological safety margin, a traffic assignment model with the addition of EVs is formulated. Subsequently, a heuristic solution algorithm is developed to solve the proposed model. Finally, the effects of travelers’ risk attitude, network degradation degree, and EV penetration rate on network performance are illustrated through an example network. The numerical results show that the difference of travelers’ risk attitudes does have impact on the route choice, and the widespread adoption of EVs can cut down the total system travel cost effectively when the transportation network is more reliable.

## Introduction

As a result of urbanization and motorization, transportation-related problems such as traffic congestion, car dependency as well as the associated noise and air pollution, have posed a major impediment to sustainable development for many contemporary cities worldwide.

Due to the advantage of high energy efficiency, low noise and zero pollution during use, electric vehicle (EV) is viewed as an effective tool to mitigate and even remedy the environmental problems resulted from transport sector. Based on this perspective, creative policies have been adopted by many cities around the world to encourage drivers to ditch conventional gasoline vehicles (GVs) in favor of clean transport. In Beijing, China, it is expected that the application scale for clean-energy vehicles will reach around 200,000 by 2017, including 180,000 battery electric vehicles (BEVs) with the supporting 5km-radius public charging service network. Therefore, there is every reason to believe that electric vehicular flow will be an important component of urban mixed traffic network in Beijing and some other similar cities all over the world.

As the last stage of traditional four-step models, traffic assignment is crucial for travel demand forecasting and transportation development plan evaluating. In essence, traffic assignment models the route choice behavior of travelers and their interactions, and the performance of a traffic assignment model relies on the behavioral assumptions behind the route choice model [1]. Given the EV cruising range limitation, the route choice behavior of EV drivers is bound to be greatly different from that of GV drivers. In addition to the traditional influencing factors like individual characteristics, route attributes and travel characteristics, energy consumption is also an important factor influencing EV drivers’ route choice. EV driver makes decision about whether charging or not and where to charge according to the cruising range and travel characteristics (such as travel distance) before the travel begins. For an EV driver, his/her route choice always comes with charging demand judgement and charging station choice, and further reshapes the traffic flow pattern on the urban transportation network.

Empirical studies reveal that travel time and monetary are the most important factors influencing route choice behavior, and travel time reliability also should not be underestimated [2], especially for the EV drivers. It is found that there is a high correlation between EV energy consumption rate and instantaneous speed from microscopic point of view [3]. Further, an EV energy consumption factor model based on the link average speed at the meso level was proposed [4]. Since the link length is fixed, it can be deduced that EV energy consumption is influenced by travel time significantly. The aging transportation infrastructure, the implementation of traffic control measures, as well as the occurrence of natural disasters and traffic incidents, are more likely to degrade the transportation network capacity entirely or locally [5], and further increase the travel time. Consequently, there will be a certain bias in travel cost for travelers, especially for the EV drivers who are more sensitive to this bias. For instance, the trip that usually can be finished without charging under normal conditions will generate the charging demand and then is changed to reach the destination in the degradable transportation network (i.e., a network with degradable link capacities, which may be subject to stochastic variations resulted from various uncertainties). In this situation, the reliability not only refers to variations in travel time, but also variations in charging demand when it comes to EVs.

Therefore, it is necessary to analyze the impact of reliability on route choice behavior, and further evaluate the performance of transportation network under different travelers’ risk attitudes, different network degradation degrees and different EVs’ penetration rates. However, the existing study on traffic assignment mainly aims at the gasoline vehicular flow, the according research achievements cannot be applied directly to the network equilibrium with the addition of EVs. Meanwhile, although the uncertainty of urban transportation network has been taken into consideration among reliability-based traffic assignment models, almost all of which focus on one unique criterion, i.e., travel time, while the equally important monetary criterion is ignored.

Different from emphasizing travel time-related criteria unilaterally, this paper comprehensively takes the travel time (including route travel time and EV charging time) and the monetary expense (including gasoline expense of GVs and electricity expense of EVs) into account to solve the mixed traffic assignment problem in degradable transportation network with the addition of EVs. In this paper, we carry the work of predecessors a major step forward by proposing a reliability-based network equilibrium framework for capturing travelers’ route choice behavior and the induced constrains with the addition of EVs. The contribution of this paper to the current literature lies in two aspects. From the practical standpoint, this paper stresses the sensibility of EVs to traffic conditions, and aims to provide some valuable suggestions for the transportation system operation and management, as well as the infrastructure planning and investment through investigating the impacts of transportation network uncertainty and EV penetration on the distribution of travel demand on transportation network and the energy demand on power network. From the theoretical standpoint, this study advances the traffic assignment research by addressing a reliability-based network equilibrium framework for accommodating urban mixed gasoline and electric vehicular flows, especially in the degradable transportation network. Particularly, a nested modelling structure is employed to cope with the route choice behavior difference derived by the different uncertainty degrees between the routes with and without degradable road links. And considering the unique energy consumption characteristics of EVs, the flow-dependent energy consumption cost and travel time, the potential en-route charging demand and charging time are all given fully consideration in this degradable transportation network research.

The outline of this paper is as follows: Section 2 reviews recent relevant literature. Section 3 describes the distributions of route travel cost components respectively. The model formulation and specifications are illustrated in Section 4, and a heuristic solution algorithm is developed to solve the model. Section 5 presents the numerical results on an example network to validate the proposed model and solution algorithm. Finally, the conclusions of the present paper are provided in Section 6.

## Literature review

A traffic assignment model is able to reflect travelers’ route choice behavior through a range of devices. Traditional assignment techniques can be classified into two categories: the Wardrop user equilibrium (UE) model and the stochastic user equilibrium (SUE) model according to the criterion that if there is a variable perception of travel costs. On the basis of these pioneering works, many theoretical models and empirical researches have been conducted to supplement and improve the behavioral assumptions behind travelers’ route choice, focusing on the time-variability of travel demand [6], the heterogeneity of travellers [7], the multi-modal characteristics of transportation network [8], and the combination of the above issues [9].

Given the gradual maturity of EV technologies and the expeditiously rising prices of crude oil, as well as a variety of government incentives and policies, the proportion of electric vehicular flow in urban mixed traffic cannot be overlooked in the near future. However, due to the unique energy consumption characteristics of EVs, the methodologies employed and the conclusions obtained in the above studies are no longer suitable for the traffic assignment problems with the addition of EVs. For example, whether a route is usable for an EV driver depends on the EV cruising range limitation and the energy consumption requirement to reach his/her destination, while all the routes on urban transportation network are usable for a GV driver in theory. A number of works can be found that explicitly incorporate EVs into traffic assignment models and accordingly evaluate the network performance. Some equilibrium models were formulated to investigate the impacts of EVs’ cruising range and recharging requirement on travels’ route choice [10]. However, the research objective is limited to pure electric vehicular flow, the interaction between the mixed traffic flows has not been mentioned. Aiming at the mixed gasoline and electric vehicular flows, the spatial distribution of travel demand of the mixed traffic flows and their corresponding travel choices are modelled further by two studies [11–12]. More specifically, the former analyzed the joint mode and route choices under the assumption that travelers have accessibility to both GVs and BEVs while the latter examined how the transportation network changes with the destination, route and parking choice adjustments by EV drivers under the assumption that the mode split is fixed. It should be noted that, both of two papers all ignored possible availability of battery-charging stations en route, and the EV energy consumption is assumed to be determined by the driving distance regardless of the traffic flows. Furthermore, some studies were presented to solve the network user equilibrium problems for the mixed battery electric vehicles and gasoline vehicles subject to the emerging charging technologies (e.g., battery swapping [13], wireless recharging [14], etc.). However, the feasibility of these new charging technologies has not been tested by the reality, and their realizations still need a long time. Gardner et al. [15] have addressed the relationship between travel demand uncertainty and EV energy consumption for the first time. In their opinion, travel time variability, which is resulted from demand uncertainty, has an influence on EV energy consumption, and further affects travelers’ route choice in user level and regional energy demand in system level. Nevertheless, the traffic assignment analysis was still based on the simplified assumptions, i.e., EVs were charged at home only and EV drivers behaved in the same manner as non-EV drivers.

In addition to stressing on the demand uncertainty in a traffic assignment context, remarkable progress has been made in the field of reliability-based user equilibrium models from the supply perspective. Given the link capacity degradation, Lo and Tung [16] formulated a probabilistic user equilibrium (PUE) model to characterize the route choice behavior, aiming to improve the travel time reliability rather than to reduce the mean travel times. This approach was extended further by Lo et al. [5] by adopting the concept of travel time budget (TTB), which is defined as the sum of the expected travel time and a safety margin to hedge against variations of travel time. The safety margin is the product of the travel time standard deviation and a scalar called punctuality parameter specified by the traveler to represent his/her risk preference. Considering the doubly uncertainties from both travel demand and network supply, Siu and Lo [17] developed a framework to model travelers’ behavior in trip planning. They claimed that all the factors that lead to the travel time variations should be taken into careful consideration, thus the notion of travel time budget plays a more relevant and important role in travelers’ travel choice compared to the notion of travel time. Later, Nie [18] pointed out that the distribution of random link capacity is more reasonable to be flow-dependent, and a percentile user equilibrium model was proposed to incorporate this viewpoint. Moreover, the percentile route travel time was evaluated by directly convolving link travel time distributions instead of relying on the central limit theorem in TTB model. Not only focusing on the reliability aspect of travel time variability unilaterally, a mean-excess traffic equilibrium (METE) model put forward by Chen and Zhou [19] also accounted for the travel time unreliability. Through hypothesizing that all travelers are willing to minimize their travel risk measured by the conditional expectation of the excess travel time for a certain travel time budget, the questions of ‘‘how much time do I need to allow?” and ‘‘how bad should I expect from the worse cases?” can be answered at the same time. More recently, Wang et al. [2] proposed a general travel time reliability bi-objective user equilibrium model to minimize both expected travel time and travel time budget. A used route with a lower expected travel time but higher variability will be less attractive than another route with a higher expected travel time but lower variability although they have the same travel time budget. In addition, based on the degradable transportation network equilibrium analysis, a series of studies were proposed to aid in the formulation and evaluation of related traffic management policies. For example, Yan et al. [20] adopted the travel time budget as travelers’ route choice principle to study the traffic assignment problem related to speed limit in a degradable transport network. The relationship among the speed limit, link capacity, and the travel time was investigated. And a user equilibrium model was established and solved to assist in the speed limit design problem. Yao et al. [21] proposed a bi-modal user equilibrium model based on the travel time budget to evaluate the exclusive bus lanes (EBLs) setting scheme in a stochastic degradable network with car and bus transit modes. Specifically, travelers by car only care about the uncertainty in terms of link travel time while travels by bus transit care about the uncertainty in terms of link travel time and waiting time at the bus stop at the same time. Liu and Huang [22] presented a user equilibrium model based on robust effective path travel time to capture travelers’ route choice behaviors influenced by the travel time uncertainty in a random degradable transportation network. The robust effective path travel time in their study is defined as combination of mean travel time and the safety margin supremum, which is similar to the concept of travel time budget. Besides, the congestion pricing design problem was investigated based on this model, and the importance of robustness on the link tolls in degradable transportation networks was revealed in their study. However, the aforementioned reliability-based user equilibrium models are all specific to the conventional transportation network without the addition of EVs, and the route choice criteria are merely related to the travel time (expected travel time, travel time variance and standard deviation). It should note that network reliability is of utmost importance for an EV driver to estimate his/her travel time and the associated travel expense, as well as to determine his/her charging demand en route.

As stated above, the popularization of EVs enriches the composition of travel cost, and meanwhile travelers’ travel cost bias resulted from network supply uncertainty leads to significant variations in travel demand on transportation network as well as the energy demand on power network. But these problems cannot be solved completely through the existing traffic assignment models and methods. Therefore, this paper attempts to integrate the gasoline and electric vehicular flow into a unified reliability-based network equilibrium framework. First, on the basis of the concept of TTB, the route travel cost budgets for GVs and EVs are calculated respectively. Then, considering a nested route choice structure, the SUE conditions are presented and formulated as an equivalent variational inequality (VI) model, which can be solved based on the method of successive weighted averages (MSWA). Finally, the impacts of travelers’ risk attitude, EV penetration rate and transportation network uncertainty on network performance are assessed through an example network.

## Travel cost budget function

Understanding the variations of travel cost components is an essential first step to estimate the generalized route travel cost in degradable transportation network. This section describes these variations for GV and EV drivers respectively. Notations used throughout the paper are provided first, followed by the distributions of travel cost components.

### Notations

Consider a strongly connected transportation network *G* (*N*, *A*), where *N* denotes the set of nodes and *A* the set of links. A limited number of charging stations are located at certain nodes of the network, and thus the usable route set for an EV with the charging demand only covers the route deployed with at least one charging station. Besides, the fast charging mode is adopted in charging stations. Let *R* and *S* represent the sets of origins and destinations respectively. *K*_*rs*_ indicates the set of all routes between the origin *r* ∈ *R* and destination *s* ∈ *S*, while $K_{rs}^{G}$ for GVs and $K_{rs}^{E}$ for EVs. $M_{k}^{rs,E}$ is the set of all the links between the origin *r* ∈ *R* and destination *s* ∈ *S* on route $k\in K_{rs}^{E}$ and $M_{k}^{rs,G}$ is that on route $k\in K_{rs}^{G}$. $M_{k}^{\prime rs,E}$ is the set of links from origin *r* ∈ *R* to charging station on route $k\in K_{rs}^{E}$.

For discussion convenience, the notations of variables and parameters used in this paper are given as follows unless specified otherwise, where superscripts *G* and *E* are used to indicate variables or parameters associated with gasoline and electric vehicles, respectively.

**Table:** 

*ξ*	EV penetration rate,%
*α*	Value of time, CNY/min
*τ*_1_	Electricity price, CNY/kwh
*τ*_2_	Gasoline price, CNY/kg
*χ*	A binary parameter whose value is 1 if an EV needs to be charged, otherwise 0
*T*_*a*_	Travel time on link *a* ∈ *A*, min
$T_{k}^{rs}$	Travel time on route *k* ∈ *K*_*rs*_ between OD pair (*r*,*s*), min
$t_{a}^{0}$	Free-flow travel time on link *a* ∈ *A*, min
*T*_0_	Charging time for a dead battery to be fully charged under fast charging mode, min
*x*_*a*_	Flow on link *a* ∈ *A*
$x_{a}^{E}$	EV flow on link $a\in M_{k}^{rs,E}$
$x_{a}^{G}$	GV flow on link $a\in M_{k}^{rs,G}$
$f_{km}^{rs,E}$	EV flow on route $k\in K_{rs}^{E}$ between OD pair (*r*,*s*) in class *m*
$f_{km}^{rs,G}$	GV flow on route $k\in K_{rs}^{G}$ between OD pair (*r*,*s*) in class *m*
*C*_*a*_	Capacity of link *a* ∈ *A*, pcu/min
${\bar{c}}_{a}$	Maximum or design capacity of link *a* ∈ *A*
*θ*_*a*_	Maximum degradable coefficient of capacity for link *a* ∈ *A*
$\delta_{a,k}^{rs}$	Route-link incidence parameter, $\delta_{a,k}^{rs}=1$ if link *a* belongs to route *k* and 0 otherwise
$e_{a}^{E}$	Energy consumption of an EV on link $a\in M_{k}^{rs,E}$, kwh
*l*_*a*_	Distance of link *a* ∈ *A*, km
*v*_*a*_	Average vehicle speed on link *a* ∈ *A*, km/h
$EF_{a}^{G}$	GV energy consumption factor on link $a\in M_{k}^{rs,G}$, kg/100km
$e_{a}^{G}$	Energy consumption of a GV on link $a\in M_{k}^{rs,G}$, kg
$e_{k}^{rs,E}$	Energy consumption of an EV on route $k\in K_{rs}^{E}$ between OD pair (*r*,*s*), kwh
$e_{ck}^{rs,E}$	Energy consumption of an EV before charging between OD pair (*r*,*s*), kwh
$e_{k}^{rs,G}$	Energy consumption of a GV on route $k\in K_{rs}^{G}$ between OD pair (*r*,*s*), kg
*S*_0_	Initial battery state of charge at origin of an EV, %
*S*′	Battery state of charge before charging, %
$T_{ck}^{rs}$	Charging time of an EV on route $k\in K_{rs}^{E}$ between OD pair (*r*,*s*), min
$C_{k}^{rs,E}$	Generalized travel cost of an EV on route $k\in K_{rs}^{E}$ between OD pair (*r*,*s*), CNY
$C_{k}^{rs,G}$	Generalized travel cost of a GV on route $k\in K_{rs}^{G}$ between OD pair (*r*,*s*), CNY
$b_{km}^{rs,E}$	Travel cost budget of an EV on route $k\in K_{rs}^{E}$ between OD pair (*r*,*s*), CNY
$b_{km}^{rs,G}$	Travel cost budget of a GV on route $k\in K_{rs}^{G}$ between OD pair (*r*,*s*), CNY
*ρ*_*m*_	Probability that a trip arrives within the travel time cost
*λ*_*m*_	A parameter related to degree of risk-aversion in class *m*
$\gamma_{a}^{E}$	Coal equivalent consumption of an EV on link $a\in M_{k}^{rs,E}$, kg
$\gamma_{a}^{G}$	Coal equivalent consumption of a GV on link $a\in M_{k}^{rs,G}$, kg

### Travel cost components

Travel cost consists of in-vehicle travel time, and the operating cost determined by the vehicle energy consumption. Besides, there is a charging demand for an EV driver when the battery state of charge (SOC) is lower than a threshold specified by the automaker. Therefore, the charging time for an EV with charging demand also should be included.

It should be noted that EVs have two types of charging modes in China and some other countries currently: slow charging and fast charging (also known as quick or rapid charging). Particularly, slow charging usually requires six to eight hours to completely charge a fully depleted battery [23] and can be performed at home or office from a standard household electrical outlet. While fast charging usually provides an 80% charge in 30 min and needs to be performed at charging stations as the emergency service [24]. Because of forgetting to charge or having been used, the SOC of BEV is not always 100% when the travel begins, thus the related limited cruising range usually causes a higher possibility of en-route charging demand to complete the entire trip [25]. Under these situations, the EVs need to be fast-charged during the trip since it can satisfy the short-term charging demand of EVs. This behavior is also similar to that of a conventional gasoline vehicle user who can refuel the vehicle at any gas stations and any time [26]. Therefore, we focus on the en-route charging demand of EVs since the network reliability is more important for an EV driver to estimate his/her travel time, which further has an impact on EV driver’s en-route charging decision. The charging behavior at the destination of the route has not been taken into consideration in this paper.

**Fig 1:**
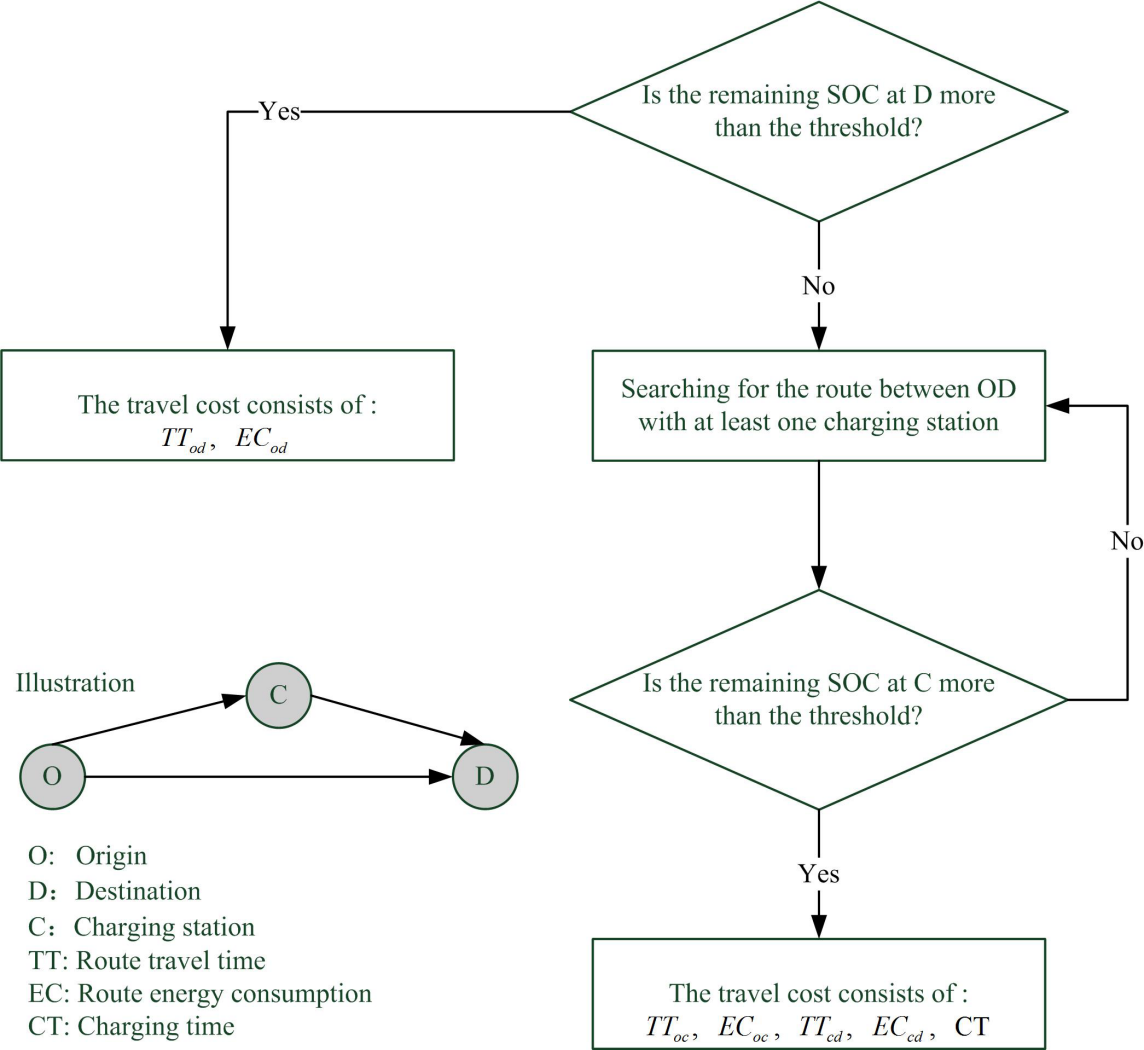
Travel cost components for EVs.

Fig 1 depicts different travel cost components for EVs with and without charging demand. Specifically, before the travel begins, the EV driver will estimate whether the vehicle can complete the trip without charging, and whether the remaining battery SOC at destination *D* is more than the threshold specified by the automaker based on the current traffic conditions. If so, the EV need not be charged en route, and the corresponding travel cost consists of the route travel time *TT*_*od*_ and the route energy consumption *EC*_*od*_ between the OD pair. If not, the EV has a demand of en-route charging. The route covering at least one charging station will be selected, and the EV driver will estimate whether the vehicle can arrive at the charging station with acceptable SOC left (i.e., more than the SOC threshold). If so, the selected route will be regarded as the usable route for EVs with charging demand, and the corresponding travel cost consists of the route travel time *TT*_*oc*_ and the route energy consumption *EC*_*oc*_ between the origin *O* and the charging station *C*, the route travel time *TT*_*cd*_ and the route energy consumption *EC*_*cd*_ between the charging station *C* and the destination *D*, as well as the charging time *CT* at the charging station *C*. If not, the selected route will be deleted from the usable route set of EVs with charging demand, and another search for usable route will be conducted until all the usable routes are selected. The following subsections describe the distributions of these cost components in degradable transportation network.

#### Link travel time

Among the degradable transportation network research, the study of Lo and Tung [16] is the most representative, and the link travel time distribution model proposed by them is also the most classical and most widely used model. Since this study is illustrative only, we conduct our study based on the work of predecessors. For sake of completeness, some of the results on link travel time distributions as derived in Lo and Tung [16] are restated as follows.

The bureau of public roads (BPR) link performance function is adopted in this study.

$$T_{a}\left(x_{a},C_{a}\right)=t_{a}^{0}\left[1+\beta {\left(\frac{x_{a}}{C_{a}}\right)}^{n}\right]$$ (1)

where *β*,*n* are the deterministic parameters. As capacity *C*_*a*_ is a random variable, so is link travel time *T*_*a*_.

Then the mean and variance of *T*_*a*_ can be written as:

$$E\left(T_{a}\right)=E\left(t_{a}^{0}\right)+\beta t_{a}^{0}E\left[{\left(\frac{x_{a}}{C_{a}}\right)}^{n}\right]=t_{a}^{0}+\beta t_{a}^{0}x_{a}^{n}E\left(\frac{1}{C_{a}^{n}}\right)$$ (2)

$$var\left(T_{a}\right)=var\left(t_{a}^{0}\right)+\beta^{2}{\left(t_{a}^{0}\right)}^{2}var\left[{\left(\frac{x_{a}}{C_{a}}\right)}^{n}\right]=\beta^{2}{\left(t_{a}^{0}\right)}^{2}x_{a}^{2n}var\left(\frac{1}{C_{a}^{n}}\right)$$ (3)

Suppose the capacity degradable random variable *C*_*a*_ is independent of the amount of traffic on it, and follows a uniform distribution defined by an upper bound ${\bar{c}}_{a}$ and a lower bound $\theta_{a}{\bar{c}}_{a}$ similar to the assumptions made in other previous studies (the generalization of the capacity random variable as a function of the link flow can be found in Lo and Tung [16]). The mean and variance of $\frac{1}{C_{a}^{n}}$ can be derived as the followings:

$$E\left(\frac{1}{C_{a}^{n}}\right)=\int_{\theta_{a}{\bar{c}}_{a}}^{{\bar{c}}_{a}}\frac{1}{C_{a}^{n}}\cdot \frac{1}{{\bar{c}}_{a}-\theta_{a}{\bar{c}}_{a}}dC_{a}^{n}=\frac{1-\theta_{a}^{1-n}}{{\bar{c}}_{a}^{n}(1-\theta_{a})(1-n)}$$ (4)

$$E\left(\frac{1}{C_{a}^{2n}}\right)=\int_{\theta_{a}{\bar{c}}_{a}}^{{\bar{c}}_{a}}\frac{1}{C_{a}^{2n}}\cdot \frac{1}{{\bar{c}}_{a}-\theta_{a}{\bar{c}}_{a}}dC_{a}^{n}=\frac{1-\theta_{a}^{1-2n}}{{\bar{c}}_{a}^{2n}(1-\theta_{a})(1-2n)}$$ (5)

$$var\left(\frac{1}{C_{a}^{n}}\right)=E\left(\frac{1}{C_{a}^{2n}}\right)-{\left[E(\frac{1}{C_{a}^{n}})\right]}^{2}=\frac{1-\theta_{a}^{1-2n}}{{\bar{c}}_{a}^{2n}(1-\theta_{a})(1-2n)}-{\left[\frac{1-\theta_{a}^{1-n}}{{\bar{c}}_{a}^{n}(1-\theta_{a})(1-n)}\right]}^{2}$$ (6)

Based on Eqs (2–6), the mean and variance of *T*_*a*_ are, respectively:

$$E\left(T_{a}\right)=t_{a}^{0}+\beta t_{a}^{0}x_{a}^{n}\frac{1-\theta_{a}^{1-n}}{{\bar{c}}_{a}^{n}(1-\theta_{a})(1-n)}$$ (7)

$$var\left(T_{a}\right)=\beta^{2}{\left(t_{a}^{0}\right)}^{2}x_{a}^{2n}\left\{\frac{1-\theta_{a}^{1-2n}}{{\bar{c}}_{a}^{2n}(1-\theta_{a})(1-2n)}-{\left[\frac{1-\theta_{a}^{1-n}}{{\bar{c}}_{a}^{n}(1-\theta_{a})(1-n)}\right]}^{2}\right\}$$ (8)

Since the capacity variable *C*_*a*_ is assumed independent, the route travel time variable $T_{k}^{rs}$ can thereby be expressed by summing the corresponding link travel time variables:

$$T_{k}^{rs}=\sum_{a}\delta_{a,k}^{rs}T_{a}$$ (9)

As assumed that link capacity distributions are independent, the mean and variance of $T_{k}^{rs}$ can be obtained as:

$$E\left(T_{k}^{rs}\right)=\sum_{a}\delta_{a,k}^{rs}E\left(T_{a}\right)$$ (10)

$$var\left(T_{k}^{rs}\right)=\sum_{a}\delta_{a,k}^{rs}var\left(T_{a}\right)$$ (11)

#### Energy consumption

In order to quantify the impact of link capacity degradation on energy consumption for EVs and GVs, it is necessary to compute the energy consumption in link and route levels.

As introduced before, EV energy consumption is influenced by travel time significantly. Since the link travel time is estimated by BPR link performance function based on the link traffic flow, it can be concluded that the EV link energy consumption is also dependent on the link traffic flow. Allowing for flow-dependent energy consumption, the EV link energy consumption function is adopted as Eq (12) proposed by He et al. [10].

$$e_{a}^{E}=0.108l_{a}+0.072T_{a}$$ (12)

Using Eqs (7) and (8), the mean and variance of EV link energy consumption are obtained as follows:

$$\begin{array}{l} E\left(e_{a}^{E}\right)=E\left(0.108l_{a}\right)+E\left(0.072T_{a}\right)\\ \;=0.108l_{a}+0.072t_{a}^{0}+\frac{0.072\beta t_{a}^{0}x_{a}^{n}\left(1-\theta_{a}^{1-n}\right)}{{\bar{c}}_{a}^{n}(1-\theta_{a})(1-n)} \end{array}$$ (13)

$$\begin{array}{l} var\left(e_{a}^{E}\right)=var\left(0.108l_{a}\right)+0.072^{2}var\left(T_{a}\right)\\ \;=0.005\beta^{2}{\left(t_{a}^{0}\right)}^{2}x_{a}^{2n}\left\{\frac{1-\theta_{a}^{1-2n}}{{\bar{c}}_{a}^{2n}(1-\theta_{a})(1-2n)}-{\left[\frac{1-\theta_{a}^{1-n}}{{\bar{c}}_{a}^{n}(1-\theta_{a})(1-n)}\right]}^{2}\right\} \end{array}$$ (14)

On the other hand, based on the vehicle fuel consumption factor model estimated by Yao and Song [27], the GV link energy consumption $e_{a}^{G}$ is calculated as the followings:

$$EF_{a}^{G}=\frac{1.276\times 10^{2}}{v_{a}}+5.298$$ (15)

$$e_{a}^{G}=EF_{a}^{G}\times \frac{l_{a}}{100}$$ (16)

where the average vehicle speed *v*_*a*_ is expressed as:

$$v_{a}=\frac{60l_{a}}{T_{a}}$$ (17)

Therefore, the mean and variance of GV link energy consumption are, respectively:

$$\begin{array}{l} E\left(e_{a}^{G}\right)=E\left(\frac{5.298l_{a}}{100}\right)+E\left(\frac{1.276T_{a}}{60}\right)\\ \;=0.0530l_{a}+0.0213t_{a}^{0}+\beta t_{a}^{0}x_{a}^{n}\frac{0.0213(1-\theta_{a}^{1-n})}{{\bar{c}}_{a}^{n}(1-\theta_{a})(1-n)} \end{array}$$ (18)

$$\begin{array}{l} var\left(e_{a}^{G}\right)=0.0530^{2}var\left(l_{a}\right)+var\left(\frac{1.276T_{a}}{60}\right)\\ \;=0.000452\beta^{2}{\left(t_{a}^{0}\right)}^{2}x_{a}^{2n}\left\{\frac{1-\theta_{a}^{1-2n}}{{\bar{c}}_{a}^{2n}(1-\theta_{a})(1-2n)}-{\left[\frac{1-\theta_{a}^{1-n}}{{\bar{c}}_{a}^{n}(1-\theta_{a})(1-n)}\right]}^{2}\right\} \end{array}$$ (19)

Similar to the calculation of route travel time distributions, the mean and variance of route energy consumption $e_{k}^{rs,E}$, $e_{k}^{rs,G}$ for two types of vehicles are written as:

$$E(e_{k}^{rs,E})=\sum_{a\in M_{k}^{rs,E}}\delta_{a,k}^{rs}E\left(e_{a}^{E}\right)$$ (20)

$$var\left(e_{k}^{rs,E}\right)=\sum_{a\in M_{k}^{rs,E}}\delta_{a,k}^{rs}var\left(e_{a}^{E}\right)$$ (21)

$$E(e_{k}^{rs,G})=\sum_{a\in M_{k}^{rs,G}}\delta_{a,k}^{rs}E\left(e_{a}^{G}\right)$$ (22)

$$var\left(e_{k}^{rs,G}\right)=\sum_{a\in M_{k}^{rs,G}}\delta_{a,k}^{rs}var\left(e_{a}^{G}\right)$$ (23)

Moreover, it should be noted that we have adopted the over-simplified energy consumption functions to estimate the vehicle energy consumption for EVs and GVs. The reasons are as follows. First, a series of findings have been published in the international journals [3, 25, 27, 28] about the vehicle energy consumption estimation. In this paper, the energy consumption model establishment is not the focus of this study. Second, the energy consumption functions adopted in this study have considered the impact of link traffic flow on link energy consumption, and have the simple function form, thus it is easy and convenient to calculate the mean and variance of link energy consumption. As described above, the mean and variance of link energy consumption are based on the integral of the probability density function of link capacity, once the form of link energy consumption is too complicated, the corresponding mean and variance are hard to be obtained. Third, the above models adopted in this paper have been published in the leading journals in the field of transportation, the feasibility and applicability of these two models have been verified by the peers.

#### Charging time

The SOC, which is an indicator of the amount of usable battery energy, is the criterion for an EV driver to decide whether or not to charge his/her vehicle. And the SOC before charging can be obtained from Eq (24).

$$S^{\prime }=S_{0}-\frac{e_{ck}^{rs,E}\times 1000}{Q\times U}\times 100\%$$ (24)

where *Q* is the nominal capacity of battery, Ah, and *U* is the battery voltage, V. These variables are usually viewed as known constants in practice.

Thus, the charging time that an EV needs to wait for being fully charged is:

$$T_{ck}^{rs}=T_{0}(1-S^{\prime })$$ (25)

Similar to the calculation of route travel time distributions, the mean and variance of EV charging time are listed as follows:

$$E\left(T_{ck}^{rs}\right)=T_{0}E\left(1-S_{0}+\frac{e_{ck}^{rs,E}\times 1000}{Q\times U}\right)=T_{0}\left(1-S_{0}\right)+\frac{1000T_{0}}{Q\times U}\sum_{a\in M_{k}^{\prime rs,E}}\delta_{a,k}^{rs}E\left(e_{a}^{E}\right)$$ (26)

$$var\left(T_{ck}^{rs}\right)=T_{0}^{2}var\left(1-S_{0}+\frac{e_{ck}^{rs,E}\times 1000}{Q\times U}\right)=\frac{T_{0}^{2}\times 10^{6}}{Q^{2}\times U^{2}}\sum_{a\in M_{k}^{\prime rs,E}}\delta_{a,k}^{rs}var\left(e_{a}^{E}\right)$$ (27)

### Generalized route travel cost

Based on the above distributions of travel cost components, the generalized route travel costs for EVs and GVs can be written as:

$$C_{k}^{rs,E}=\alpha \left[T_{k}^{rs}+\chi T_{ck}^{rs}\right]+\tau_{1}e_{k}^{rs,E}$$ (28)

$$C_{k}^{rs,G}=\alpha T_{k}^{rs}+\tau_{2}e_{k}^{rs,G}$$ (29)

And their mean and standard deviation are, respectively:

$$E\left(C_{k}^{rs,E}\right)=\alpha \left[E\left(T_{k}^{rs}\right)+\chi E\left(T_{ck}^{rs}\right)\right]+\tau_{1}E\left(e_{k}^{rs,E}\right)$$ (30)

$$\begin{array}{l} \sigma \left(C_{k}^{rs,E}\right)=\sqrt{var\left(\alpha T_{k}^{rs}+\alpha \chi T_{ck}^{rs}+\tau_{1}e_{k}^{rs,E}\right)}\\ \;=\sqrt{E[{\left(\alpha T_{k}^{rs}+\alpha \chi T_{ck}^{rs}+\tau_{1}e_{k}^{rs,E}\right)}^{2}]\text{-}{\left[E(\alpha T_{k}^{rs}+\alpha \chi T_{ck}^{rs}+\tau_{1}e_{k}^{rs,E})\right]}^{2}}\\ \;=\sqrt{\begin{array}{l} \alpha^{2}\mathrm{var}(T_{k}^{rs})+\alpha^{2}\chi^{2}\mathrm{var}(T_{ck}^{rs})+\tau_{1}^{2}\mathrm{var}(e_{k}^{rs,E})+2\alpha^{2}\chi [E(T_{k}^{rs}T_{ck}^{rs})-E(T_{k}^{rs})E(T_{ck}^{rs})]\\ +2\alpha \tau_{1}[E(T_{k}^{rs}e_{k}^{rs,E})-E(T_{k}^{rs})E(e_{k}^{rs,E})]+2\alpha \chi \tau_{1}[E(T_{ck}^{rs}e_{k}^{rs,E})-E(T_{ck}^{rs})E(e_{k}^{rs,E})] \end{array}}\\ \;=\sqrt{\begin{array}{l} \alpha^{2}\mathrm{var}(T_{k}^{rs})+\alpha^{2}\chi^{2}\mathrm{var}(T_{ck}^{rs})+\tau_{1}^{2}\mathrm{var}(e_{k}^{rs,E})+2\alpha^{2}\chi \times 0.072\times \frac{1000T_{0}}{Q\times U}\sum_{a\in M_{k}^{\prime rs,E}}\delta_{a,k}^{rs}\mathrm{var}\left(T_{a}\right)\\ +2\alpha \tau_{1}\times 0.072\sum_{a\in M_{k}^{\prime rs,E}}\delta_{a,k}^{rs}var\left(T_{a}\right)+2\alpha \chi \tau_{1}\times 0.072^{2}\times \frac{1000T_{0}}{Q\times U}\sum_{a\in M_{k}^{\prime rs,E}}\delta_{a,k}^{rs}var\left(T_{a}\right) \end{array}} \end{array}$$ (31)

$$E\left(C_{k}^{rs,G}\right)=\alpha E\left(T_{k}^{rs}\right)+\tau_{2}E\left(e_{k}^{rs,G}\right)$$ (32)

$$\begin{array}{l} \sigma \left(C_{k}^{rs,G}\right)=\sqrt{var\left(\alpha T_{k}^{rs}+\tau_{2}e_{k}^{rs,G}\right)}\\ \;=\sqrt{E[{\left(\alpha T_{k}^{rs}+\tau_{2}e_{k}^{rs,G}\right)}^{2}]-{\left[E\left(\alpha T_{k}^{rs}+\tau_{2}e_{k}^{rs,G}\right)\right]}^{2}}\\ \;=\sqrt{\alpha^{2}\mathrm{var}(T_{k}^{rs})+\tau_{2}^{2}\mathrm{var}(e_{k}^{rs,G})+2\alpha \tau_{2}[E(T_{k}^{rs}e_{k}^{rs,G})-E(T_{k}^{rs})E(e_{k}^{rs,G})]}\\ \;=\sqrt{\alpha^{2}\mathrm{var}(T_{k}^{rs})+\tau_{2}^{2}\mathrm{var}(e_{k}^{rs,G})+2\alpha \tau_{2}\times \frac{1.276}{60}\sum_{a}\delta_{a,k}^{rs}var\left(T_{a}\right)} \end{array}$$ (33)

In order to capture the stochastic change of generalized route travel cost due to the stochastic perturbation on link capacity in degradable transportation network, based on the concept of travel time budget (TTB), the route choice principle for EV and GV drivers can be expressed as follows:

$$b_{km}^{rs,i}=E\left(C_{k}^{rs,i}\right)+\lambda_{m}\sigma \left(C_{k}^{rs,i}\right)\;i=E,G$$ (34)

According to the Central Limit Theorem, when the number of links with nonzero variance is large enough for a route, the generalized route travel cost follows a normal distribution. As a result, *λ*_*m*_ has a close relation with the probability *ρ*_*m*_ that a trip arrives within the travel cost budget, and can be written as:

$$P\left\{C_{k}^{rs,i}\leq b_{km}^{rs,i}=E\left(C_{k}^{rs,i}\right)+\lambda_{m}\sigma \left(C_{k}^{rs,i}\right)\right\}=\rho_{m}\;i=E,G$$ (35)

Rearrange this equation leads to:

$$P\left(S_{C_{k}}^{i}=\frac{C_{k}^{rs,i}-E\left(C_{k}^{rs,i}\right)}{\sigma \left(C_{k}^{rs,i}\right)}\leq \lambda_{m}\right)=\rho_{m}\;i=E,G$$ (36)

where the left hand side in Eq (36) is the standard normal variate of $C_{k}^{rs,i}$, $S_{C_{k}}^{i}∼N(0,1)$, and the value of *λ*_*m*_ determined by the probability *ρ*_*m*_ influences the travel cost budget $b_{km}^{rs,i}$ as in Eq (34).

## Model formulation

In this section, the modelling process is described in details. First, a reliability-based traffic assignment model with the addition of EVs is proposed based on the route choice behavior analysis. Then, an equivalent VI problem is formulated to depict the SUE conditions. Finally, the solution algorithm used to solve the model is illustrated on the basis of the MSWA.

### Equilibrium conditions

**Fig 2:**
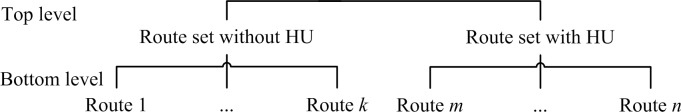
Nested route choice structure.

In this paper, it is assumed that all the travelers make their route choice decisions based on the perceived generalized travel cost distribution. Since the multinomial logit (MNL) model exhibits the independence from irrelevant alternative (IIA) property, which is resulted from independently and identically distributed (IID) assumptions inherited in using the Gumbel distributed perception errors, the MNL model has a fatal weakness when used in degradable transportation network equilibrium analysis. Specially, the variance of the Gumbel error term for routes whose capacities are not degraded is obviously different from which for routes whose capacities are degraded, i.e., these perception errors don’t follow the same Gumbel distribution. Therefore, a nested route choice structure is employed to deal with the relatively larger perceived error difference among route alternatives through grouping routes considered to have similar perception error in hierarchies or nests. As shown in Fig 2, the routes that have or don’t have high uncertainty (HU) are classified into the same nest respectively, leading to a two-level nested logit (NL) route choice model: the bottom level focuses on route choice among the route set, and further influences the top level route set choice.

The perceived generalized route travel costs for EV and GV drivers are represented as:

$$B_{km}^{rs,i}=b_{km}^{rs,i}+\varepsilon_{km}^{rs,i}\;i=E,G$$ (37)

where $\varepsilon_{km}^{rs,E}$ and $\varepsilon_{km}^{rs,G}$ denote the error terms.

And the possibilities $P_{m}^{rs,i}(wk)$ for EV drivers and GV drivers in class *m* choose route *k* between OD pair (*r*,*s*) follow the logit-based principle.

$$P_{m}^{rs,i}(wk)=P_{m}^{rs,i}(w)\cdot P_{m}^{rs,i}(k|w)\;i=E,G$$ (38)

where $P_{m}^{rs,i}(w)$ is the marginal probability of route set *w* being chosen, and $P_{m}^{rs,i}(k|w)$ is the conditional probability of route choice given that route set *w* is selected. In this two-level NL choice structure, $P_{m}^{rs,i}(k|w)$ can be expressed with a MNL expression as:

$$P_{m}^{rs,i}(k|w)=\frac{\exp(-\mu_{1}b_{km}^{rs,i})}{\sum_{l\in K_{rs}^{i,w}}\exp(-\mu_{1}b_{lm}^{rs,i})}\;i=E,G$$ (39)

And $P_{m}^{rs,i}(w)$ can be calculated by:

$$P_{m}^{rs,i}(w)=\frac{\exp(-\mu_{2}V_{wm}^{rs,i}{}^{*})}{\sum_{l\in N_{w}}\exp(-\mu_{2}V_{lm}^{rs,i}{}^{*})}\;i=E,G$$ (40)

where *N*_*w*_ is the number of alternatives on top level, $V_{wm}^{rs,i}{}^{*}$ is the natural logarithm of the denominator (i.e., log sum term) of $P_{m}^{rs,i}(k|w)$, it is viewed as the expected maximum utility of routes set *w* (i.e., $K_{rs}^{i,w}$).

$$V_{wm}^{rs,i}{}^{*}=-\frac{1}{\mu_{1}}\mathrm{In}\sum_{l\in K_{rs}^{i,w}}\exp(-\mu_{1}b_{lm}^{rs,i})\;i=E,G$$ (41)

Besides, the expected maximum utility between the OD pair (*r*,*s*) can be expressed as:

$$V_{m}^{rs,i}{}^{*}=-\frac{1}{\mu_{2}}\mathrm{In}\sum_{N_{w}}\exp(-\mu_{2}V_{wm}^{rs,i}{}^{*})\;i=E,G$$ (42)

Moreover, *μ*_1_ and *μ*_2_ are the scaling parameters for bottom and top level respectively, and can be computed as shown in Eqs (43) and (44):

$$\mu_{1}=\frac{\pi }{\sqrt{6C_{\hat{k}1}^{rs}}}$$ (43)

$$\mu_{2}=\frac{\pi }{\sqrt{6(C_{\hat{k}1}^{rs}+C_{\hat{k}2}^{rs})}}$$ (44)

where $C_{\hat{k}1}^{rs}$ and $C_{\hat{k}2}^{rs}$ can be viewed as the minimum generalized route travel cost of routes and of route sets respectively. It should note that all routes at same level are assumed to have the identical variance, because scaling each route with a different scaling factor would violate the logit-based SUE models’ assumption [29].

On this occasion, the conditions of the stochastic user equilibrium can be characterized by the following equations:

$$\{\begin{array}{ll} \;Q_{rs}=Q_{rs}^{G}+Q_{rs}^{E} & \\ \;Q_{rs}^{i}=\sum_{w}\sum_{m}f_{mw}^{rs,i} & i=E,G\\ \;Q_{rs}^{i,w}=\sum_{k\in K_{rs}^{i,w}}\sum_{m}f_{mk|w}^{rs,i} & i=E,G\\ f_{mk|w}^{rs,i}=P_{m}^{rs,i}(k|w)\cdot Q_{rs}^{i}\cdot \eta_{m} & i=E,G\\ \;f_{mw}^{rs,i}=P_{m}^{rs,i}(w)\cdot Q_{rs}^{i}\cdot \eta_{m} & i=E,G\\ \;f_{mk|w}^{rs,i}\geq 0,f_{mw}^{rs,i}\geq 0 & i=E,G\\ \;Q_{rs}^{i}\geq 0,Q_{rs}^{i,w}\geq 0 & i=E,G\\ \;x_{a}=\sum_{rs}\sum_{m}\sum_{k}\sum_{i}\sum_{w}\delta_{a,k}^{rs}f_{mk|w}^{rs,i} & \end{array}$$ (45)

where *Q*_*rs*_ is total travel demand between OD pair (*r*,*s*); $Q_{rs}^{i}$ is the travel demand between OD pair (*r*,*s*) for mode *i* (i.e., EV or GV); $Q_{rs}^{i,w}$ is the travel demand between OD pair (*r*,*s*) for mode *i* choosing route set *w*; $f_{mk|w}^{rs,i}$ is traffic flow between OD pair (*r*,*s*) for mode *i* choosing route *k* given the route set *w* is selected; $f_{mw}^{rs,i}$ is traffic flow between OD pair (*r*,*s*) for mode *i* choosing route set *w*; and *η*_*m*_ is the population proportion of class *m*, %.

### VI formulation

Mathematically, the SUE conditions (45) can be formulated as an equivalent VI problem as follows:

$$\begin{array}{l} \sum_{rs}\sum_{m}\sum_{k\in K_{rs}^{E,HU}}\left(b_{km|w}^{rs,E}\left(f_{km|w}^{rs,E*}\right)+\frac{1}{\mu_{1}}In\frac{f_{km|w}^{rs,E*}}{\eta_{m}Q_{rs}^{E,w}}-V_{wm}^{rs,E*}\right)\left(f_{km|w}^{rs,E}-f_{km|w}^{rs,E*}\right)\\ +\sum_{rs}\sum_{m}\sum_{w}\left(\frac{1}{\mu_{2}}In\frac{f_{mw}^{rs,E*}}{\eta_{m}Q_{rs}^{E}}+V_{wm}^{rs,E*}-V_{m}^{rs,E*}\right)\left(f_{mw}^{rs,E}-f_{mw}^{rs,E*}\right)\\ +\sum_{rs}\sum_{m}\sum_{k\in K_{rs}^{G,HU}}\left(b_{km|w}^{rs,G}\left(f_{km|w}^{rs,G*}\right)+\frac{1}{\mu_{1}}In\frac{f_{km|w}^{rs,G*}}{\eta_{m}Q_{rs}^{G,w}}-V_{wm}^{rs,G*}\right)\left(f_{km|w}^{rs,G}-f_{km|w}^{rs,G*}\right)\\ +\sum_{rs}\sum_{m}\sum_{w}\left(\frac{1}{\mu_{2}}In\frac{f_{mw}^{rs,G*}}{\eta_{m}Q_{rs}^{G}}+V_{wm}^{rs,G*}-V_{m}^{rs,G*}\right)\left(f_{mw}^{rs,G}-f_{mw}^{rs,G*}\right)\geq 0 \end{array}$$ (46)

where the variables with * is the decision variables; $b_{km|w}^{rs,E}$ (or $b_{km|w}^{rs,G}$) is the travel cost budget of an EV (or a GV) on route $k\in K_{rs}^{E,w}$ (or $k\in K_{rs}^{G,HU}$) between OD pair (*r*,*s*) in class *m*, CNY.

The equivalence of the VI formulation and the SUE conditions as well as the existence of an equilibrium solution are given by the following propositions.

**Proposition 1. *The VI problem(46) is equivalent to the SUE conditions(45)***.

**Proof.** The KKT (Karush-Kuhn-Tucker) conditions of the VI problem (46) are:

$$f_{km|w}^{rs,E}:\;b_{km|w}^{rs,E}\left(f_{km|w}^{rs,E}\right)+\frac{1}{\mu_{1}}In\frac{f_{km|w}^{rs,E}}{\eta_{m}Q_{rs}^{E,w}}-V_{wm}^{rs,E}=0$$ (47)

$$f_{mw}^{rs,E}:\;b_{mw}^{rs,E}\left(f_{mw}^{rs,E}\right)+\frac{1}{\mu_{2}}In\frac{f_{mw}^{rs,E}}{\eta_{m}Q_{rs}^{E}}+V_{wm}^{rs,E}-V_{m}^{rs,E}=0$$ (48)

$$f_{km|w}^{rs,G}:\;b_{km|w}^{rs,G}\left(f_{km|w}^{rs,G}\right)+\frac{1}{\mu_{1}}In\frac{f_{km|w}^{rs,G}}{\eta_{m}Q_{rs}^{G,w}}-V_{wm}^{rs,G}=0$$ (49)

$$f_{mw}^{rs,G}:\;b_{mw}^{rs,G}\left(f_{mw}^{rs,G}\right)+\frac{1}{\mu_{2}}In\frac{f_{mw}^{rs,G}}{\eta_{m}Q_{rs}^{G}}+V_{wm}^{rs,G}-V_{m}^{rs,G}=0$$ (50)

where Eqs (47) and (48) can be rewritten as:

$$\frac{f_{km|w}^{rs,E}}{\eta_{m}Q_{rs}^{E,w}}=\exp(-\mu_{1}(b_{km|w}^{rs,E}\left(f_{km|w}^{rs,E}\right)-V_{wm}^{rs,E})$$ (51)

$$\frac{f_{mw}^{rs,E}}{\eta_{m}Q_{rs}^{E}}=\exp(-\mu_{2}(b_{mw}^{rs,E}\left(f_{mw}^{rs,E}\right)+V_{wm}^{rs,E}-V_{m}^{rs,E}))$$ (52)

Combining Eq (41) with Eq (51) leads to:

$$\frac{f_{km|w}^{rs,E}}{\eta_{m}Q_{rs}^{E,w}}=\frac{\exp(-\mu_{1}b_{km|w}^{rs,E}\left(f_{km|w}^{rs,E}\right))}{\sum_{l}\exp(-\mu_{1}b_{lm}^{rs,E})}$$ (53)

which corresponds to the conditional probability of the NL model (Eq (39)).

Combining Eq (52) with Eq (42) leads to:

$$\frac{f_{mw}^{rs,E}}{\eta_{m}Q_{rs}^{E}}=\frac{\exp(-\mu_{2}V_{wm}^{rs,E})}{\sum_{l\in N_{w}}\exp(-\mu_{2}V_{lm}^{rs,E})}$$ (54)

which corresponds to the marginal probability of the NL model (Eq (40)).

Similarly, Eqs (49) and (50) can also be deduced through the above manipulations.

Therefore, it is easy to see that the proposed VI problem is equivalent to the SUE conditions. This completes the proof.

**Proposition 2. *At least one solution of the VI problem (46) exists***.

**Proof.** Assume that the travel time is the strictly monotonic increasing function of the link flow, let us set:

$$F\left(f_{km|w}^{rs,E}\right)=b_{km|w}^{rs,E}\left(f_{km|w}^{rs,E}\right)+\frac{1}{\mu_{1}}In\frac{f_{km|w}^{rs,E}}{\eta_{m}Q_{rs}^{E,w}}-V_{wm}^{rs,E}$$ (55)

$$F(f_{mw}^{rs,E})=b_{mw}^{rs,E}\left(f_{mw}^{rs,E}\right)+\frac{1}{\mu_{2}}In\frac{f_{mw}^{rs,E}}{\eta_{m}Q_{rs}^{E}}+V_{wm}^{rs,E}-V_{m}^{rs,E}$$ (56)

$$F(f_{km|w}^{rs,G})=b_{km|w}^{rs,G}\left(f_{km|w}^{rs,G}\right)+\frac{1}{\mu_{1}}In\frac{f_{km|w}^{rs,G}}{\eta_{m}Q_{rs}^{G,w}}-V_{wm}^{rs,G}$$ (57)

$$F(f_{mw}^{rs,G})=b_{mw}^{rs,G}\left(f_{mw}^{rs,G}\right)+\frac{1}{\mu_{2}}In\frac{f_{mw}^{rs,G}}{\eta_{m}Q_{rs}^{G}}+V_{wm}^{rs,G}-V_{m}^{rs,G}$$ (58)

The derivatives of the above equations with respect to path flow variables are,

$$\begin{array}{l} \frac{\partial F\left(f_{km|w}^{rs,E}\right)}{\partial f_{km|w}^{rs,E}}=\frac{\partial b_{km|w}^{rs,E}\left(f_{km|w}^{rs,E}\right)}{\partial f_{km|w}^{rs,E}}+\frac{1}{\mu_{1}}\frac{1}{f_{km|w}^{rs,E}}-\frac{\partial V_{wm}^{rs,E}}{\partial b_{km|w}^{rs,E}\left(f_{km|w}^{rs,E}\right)}\frac{\partial b_{km|w}^{rs,E}\left(f_{km|w}^{rs,E}\right)}{\partial f_{km|w}^{rs,E}}\\ \;=\frac{\partial b_{km|w}^{rs,E}\left(f_{km|w}^{rs,E}\right)}{\partial f_{km|w}^{rs,E}}+\frac{1}{\mu_{1}}\frac{1}{f_{km|w}^{rs,E}}-P_{m}^{rs,E}(k|w)\frac{\partial b_{km|w}^{rs,E}\left(f_{km|w}^{rs,E}\right)}{\partial f_{km|w}^{rs,E}}\\ \;=(1-P_{m}^{rs,E}(k|w))\frac{\partial b_{km|w}^{rs,E}\left(f_{km|w}^{rs,E}\right)}{\partial f_{km|w}^{rs,E}}+\frac{1}{\mu_{1}}\frac{1}{f_{km|w}^{rs,E}}>0 \end{array}$$ (59)

$$\begin{array}{l} \frac{\partial F(f_{mw}^{rs,E})}{\partial f_{mw}^{rs,E}}=\frac{\partial b_{mw}^{rs,E}\left(f_{mw}^{rs,E}\right)}{\partial f_{mw}^{rs,E}}+\frac{1}{\mu_{2}}\frac{1}{f_{mw}^{rs,E}}+\frac{\partial V_{wm}^{rs,E}}{\partial b_{km|w}^{rs,E}\left(f_{km|w}^{rs,E}\right)}\frac{\partial b_{km|w}^{rs,E}\left(f_{km|w}^{rs,E}\right)}{\partial f_{km|w}^{rs,E}}\frac{\partial f_{km|w}^{rs,E}}{\partial f_{mw}^{rs,E}}-\frac{\partial V_{m}^{rs,E}}{\partial b_{mw}^{rs,E}\left(f_{mw}^{rs,E}\right)}\frac{\partial b_{mw}^{rs,E}\left(f_{mw}^{rs,E}\right)}{\partial f_{mw}^{rs,E}}\\ \;=\frac{\partial b_{mw}^{rs,E}\left(f_{mw}^{rs,E}\right)}{\partial f_{mw}^{rs,E}}+\frac{1}{\mu_{2}}\frac{1}{f_{mw}^{rs,E}}+P_{m}^{rs,E}(k|w)\frac{\partial b_{km|w}^{rs,E}\left(f_{km|w}^{rs,E}\right)}{\partial f_{km|w}^{rs,E}}\frac{\partial f_{km|w}^{rs,E}}{\partial f_{mw}^{rs,E}}-P_{m}^{rs,E}(w)\frac{\partial b_{mw}^{rs,E}\left(f_{mw}^{rs,E}\right)}{\partial f_{mw}^{rs,E}}\\ \;=(1-P_{m}^{rs,E}(w))\frac{\partial b_{mw}^{rs,E}\left(f_{mw}^{rs,E}\right)}{\partial f_{mw}^{rs,E}}+\frac{1}{\mu_{2}}\frac{1}{f_{mw}^{rs,E}}+P_{m}^{rs,E}(k|w)\frac{\partial b_{km|w}^{rs,E}\left(f_{km|w}^{rs,E}\right)}{\partial f_{km|w}^{rs,E}}\frac{\partial f_{km|w}^{rs,E}}{\partial f_{mw}^{rs,E}}>0 \end{array}$$ (60)

$$\frac{\partial F\left(f_{km|w}^{rs,G}\right)}{\partial f_{km|w}^{rs,G}}=(1-P_{m}^{rs,G}(k|w))\frac{\partial b_{km|w}^{rs,G}\left(f_{km|w}^{rs,G}\right)}{\partial f_{km|w}^{rs,G}}+\frac{1}{\mu_{1}}\frac{1}{f_{km|w}^{rs,G}}>0$$ (61)

$$\frac{\partial F(f_{mw}^{rs,G})}{\partial f_{mw}^{rs,G}}=(1-P_{m}^{rs,G}(w))\frac{\partial b_{mw}^{rs,G}\left(f_{mw}^{rs,G}\right)}{\partial f_{mw}^{rs,G}}+\frac{1}{\mu_{2}}\frac{1}{f_{mw}^{rs,G}}+P_{m}^{rs,G}(k|w)\frac{\partial b_{km|w}^{rs,G}\left(f_{km|w}^{rs,G}\right)}{\partial f_{km|w}^{rs,G}}\frac{\partial f_{km|w}^{rs,G}}{\partial f_{mw}^{rs,G}}>0$$ (62)

It can be easily seen that $F\left(f_{km|w}^{rs,E}\right)$, $F(f_{mw}^{rs,E})$, $F(f_{km|w}^{rs,G})$ and $F(f_{mw}^{rs,G})$ are the strictly monotone increasing functions with respect to path flows. Moreover, since being composed of nonnegative linear constraints, the feasible region of the VI problem is compact and convex. Thus, the uniqueness of solution can be guaranteed. This completes the proof.

### Solution algorithm

A number of methods can be used to solve the VI problem. Compared with optimization algorithm, the heuristic solution algorithm is more practical and efficient, it can find an approximate suitable stepsize without evaluating the complex objective function, and finally converges to the optimal solution through several iterations. Among the heuristic solution algorithm, the MSWA is widely used to solve the stochastic user equilibrium problems [30–32]. This paper solves the problem by a heuristic solution algorithm developed from the MSWA. Specific steps are as follows:

**Step 1.** Initialization. Input the network characteristics and set the initial link flows *x*_*a*_. Meanwhile, let the iteration *n* = 1.
**Step 2.** Distribution update.
  - **Step 2.1:** Update the means and variances of link travel time based on Eqs (7) and (8); the means and variances of link energy consumption for EVs and GVs, respectively, based on Eqs (13), (14), (18) and (19).
  - **Step 2.2:** Calculate the means and variances of route travel time via Eqs (10) and (11); the means and variances of GV route energy consumption via Eqs (22) and (23); the means and variances of EV route energy consumption, including which on the routes that an EV from the origin to the destination and on the routes that an EV from the origin to the charging station, via Eqs (20) and (21).
  - **Step 2.3:** Adjust the usable route set for EV drivers. If the initial usable battery energy of an EV can meet the energy requirement, then the usable route set is the same as the GV drivers. If not, the usable route set must cover at least one charging station, and ensure the EV can reach the charging station also with acceptable SOC left.
  - **Step 2.4:** Using Eqs (26) and (27), calculate the means and variances of EV charging time. And update the generalized route travel cost budgets in class *m* via Eqs (30–34).
**Step 3.** Search Direction. According to the route choice probability formulas (Eqs (38–40) and Eq (63) below, obtain the auxiliary flows on routes $f_{km}^{rs,i}$ and links $x_{a}^{\prime }$.
  $$x_{a}^{\prime }=\sum_{rs}\sum_{m}\sum_{k}\sum_{i}\delta_{a,k}^{rs}f_{km}^{rs,i}$$ (63)
**Step 4.** Iteration. Update the link flows by the MSWA.
  $${\left(x_{a}\right)}^{n+1}={\left(x_{a}\right)}^{n}+\frac{1}{1+2+\cdots n}[{\left(x_{a}^{\prime }\right)}^{n}-{\left(x_{a}\right)}^{n}]$$ (64)
**Step 5.** Convergence Check. If the merit function *F* satisfies the following formulation:
  $$F=\frac{\sqrt{\sum_{a\in A}{\left({\left(x_{a}\right)}^{n+1}-{\left(x_{a}\right)}^{n}\right)}^{2}}}{\sum_{a\in A}{\left(x_{a}\right)}^{n}}\leq \psi$$ (65)

then stop, where *ψ* is the convergence criterion for *F*; otherwise let *n* = *n* + 1, return to Step 2.

## Numerical example

In this section, a set of numerical and computational analysis results with the proposed model are presented to illustrate the effects of considering factors, including travelers’ risk attitude toward network uncertainty, the network degradation degree, and the penetration rate of EVs.

**Fig 3:**
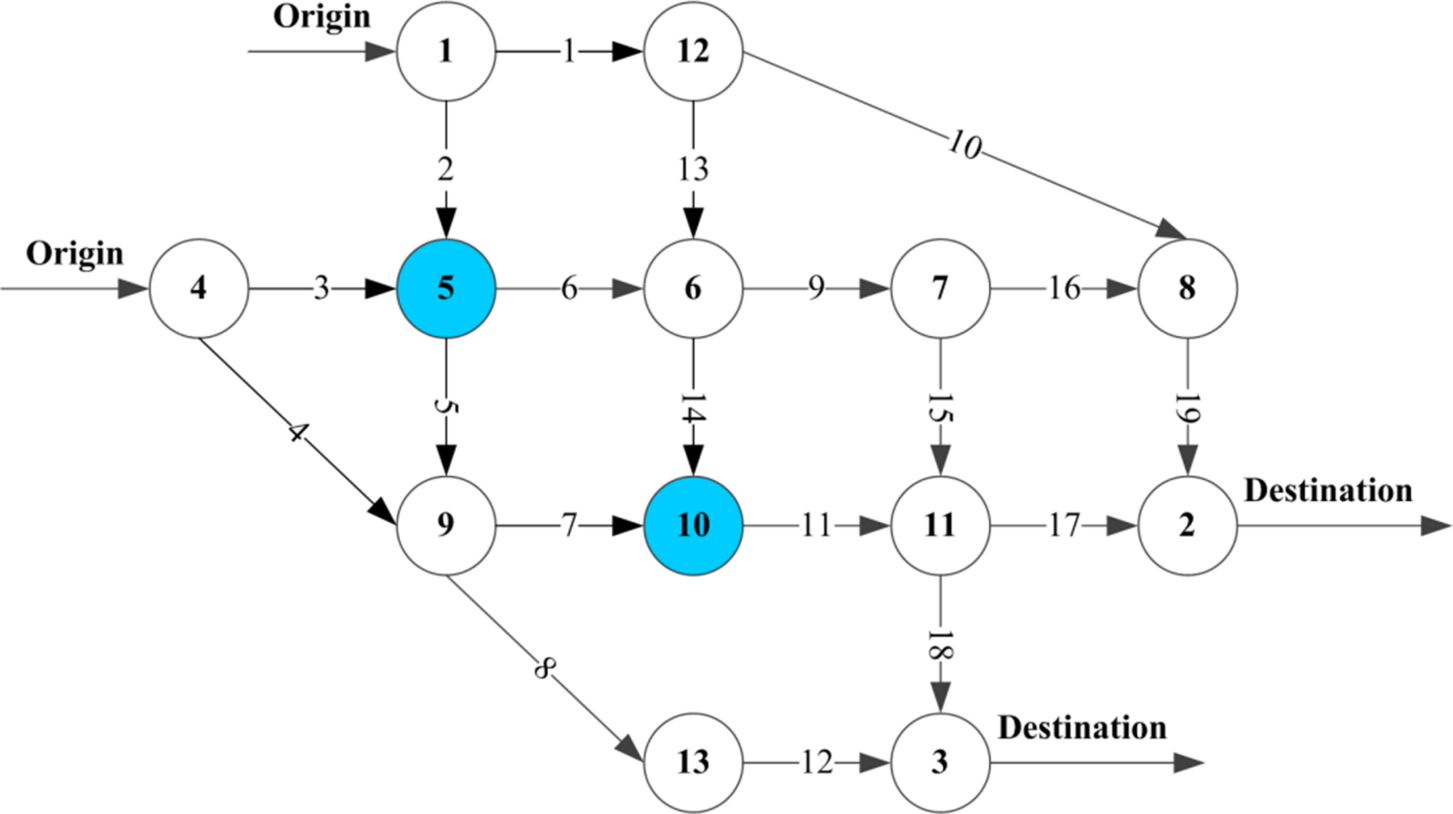
The Nguyen–Dupius network.

The Nguyen–Dupius network shown in Fig 3 is adopted as an illustrative example. It consists of four OD pairs, 19 links, and 13 nodes, where nodes 5 and 10 represent the public charging stations. The link characteristics can be found in Nguyen and Dupius [33]. The free-flow speed on all links is set as 60 km/h, and thus the link distance of each link is approximated as its free-flow travel time. In addition, a threshold of 30% SOC is adopted in this paper according to the suggestion by the Beijing Automotive Industry Corp (BAIC). The link performance function (Eq (1)) is with *β* = 1, *n* = 4. Additional parameter values such as the value of time *α* = 0.478 CNY/min, electricity price *τ*_1_ = 0.488 CNY/kwh, gasoline price *τ*_2_ = 9.076 CNY/kg, and charging time for a dead battery to be fully charged under fast charging mode *T*_0_ = 37.5 min are suggested according to the general situations in Beijing, China. It should be noted that this paper focuses on a long-term network equilibrium pattern. The inputs and outputs are all at a mean level. For the input parameter related to the charging time at the charging station, it can be viewed as the average charging time including the waiting or searching time and the actual charging time. The queuing at the charging station hasn’t been taken into account in this study.

Moreover, suppose there are three classes of travelers with different degrees of risk-aversion on the transportation network, namely low reliability (LR), medium reliability (MR) and high reliability (HR) travelers. And EV drivers in the same class have the equal initial battery SOC at their origins. Based on the survey data collected by Lo et al. [5], the parameters *λ*_*m*_ are set as 0.1, 0.86, 1.72, corresponding to the within budget cost reliability of 54%, 81% and 96%, respectively. The respective population proportions of these three classes of travelers are 49.5%, 38% and 12.5%. Besides, the initial battery SOC for LR, MR and HR EV drivers are set as 50%, 60% and 70%, respectively given the risk-aversion degrees, i.e., the lower reliability the driver is with, the less initial battery SOC the EV has, leading to a shorter driving range.

Table 1 enumerates the travel demands for all O–D pairs and all effective paths of the example network, where 1(1-10-19) represents that route 1 is comprised by links 1, 10, and 19.

**Table 1:** OD demands and route composition.

OD pairs	(1, 2)	(1, 3)	(4, 2)	(4, 3)
**Travel demands**	400	800	600	200
**Routes**	1(1-10-19)	1(2-5-8-12)	1(4-7-11-17)	1(4-8-12)
	2(2-6-9-16-19)	2(2-6-9-15-18)	2(3-6-9-16-19)	2(4-7-11-18)
	3(2-6-9-15-17)	3(2-6-14-11-18)	3(3-6-9-15-17)	3(3-5-8-12)
	4(2-6-14-11-17)	4(2-5-7-11-18)	4(3-6-14-11-17)	4(3-6-9-15-18)
	5(2-5-7-11-17)	5(1-13-9-15-18)	5(3-5-7-11-17)	5(3-6-14-11-18)
	6(1-13-9-16-19)	6(1-13-14-11-18)		6(3-5-7-11-18)
	7(1-13-9-15-17)			
	8(1-13-14-11-17)			

**Fig 4:**
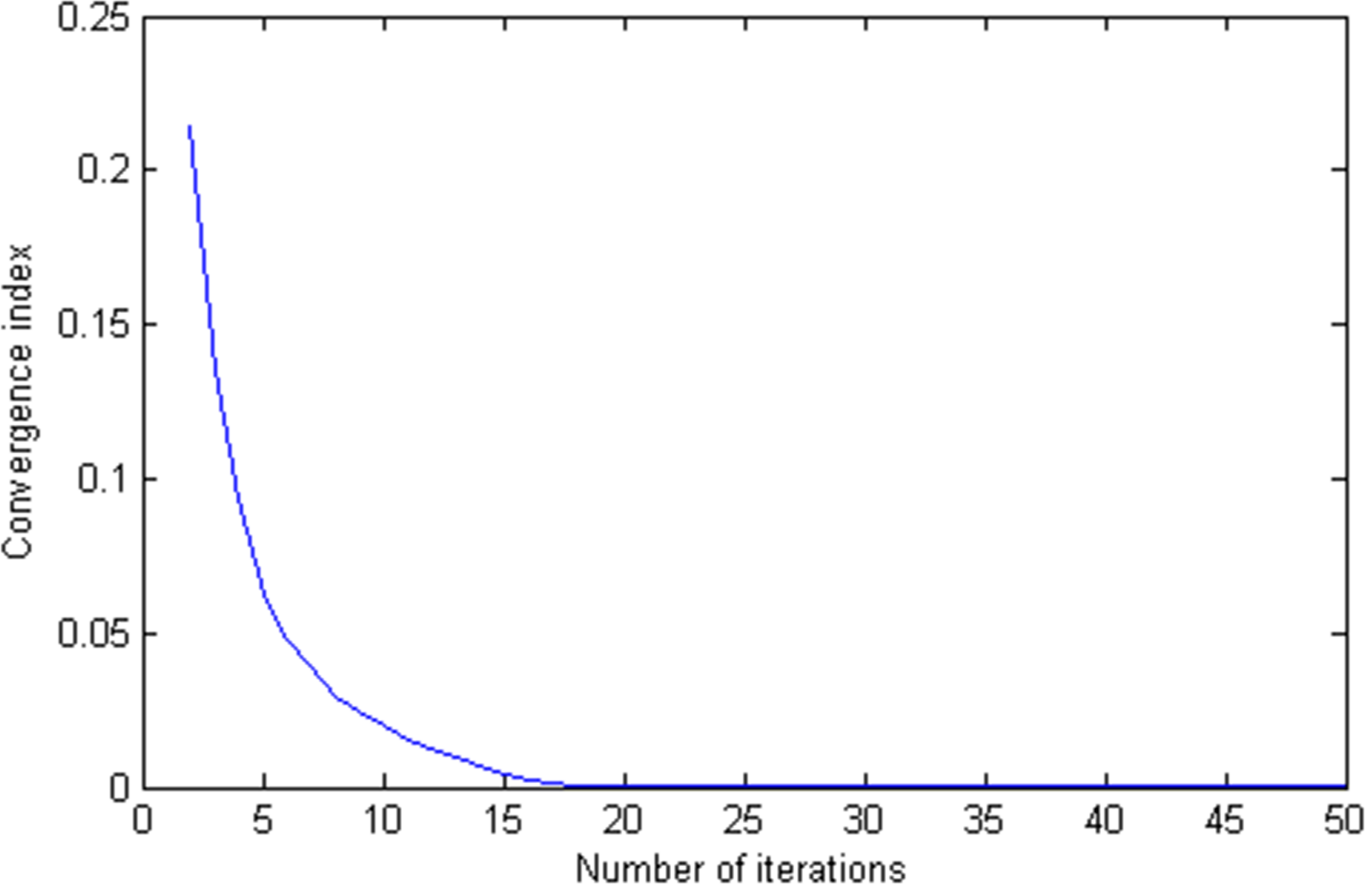
Convergence of the solution algorithm.

In the case of maximum degradable coefficient *θ*_*a*_ = 0.7 for all links (i.e., moderate degradation of the entire transportation network), and EV penetration rate *ξ* = 20% (i.e., the smaller EV market share), the convergence performance of the MSWA-based algorithm is shown in Fig 4 and Table 2.

**Table 2:** Change process of convergence index and link flow.

Convergence step		1	5	10	15	20	25	30
**Link flow**	1	124	382	396	410	412	413	413
	2	1076	818	804	790	788	787	787
	3	640	445	435	444	443	443	443
	4	160	355	365	356	357	357	357
	5	149	377	472	436	439	438	438
	6	1567	887	767	798	792	792	792
	7	47	266	254	239	238	238	238
	8	262	466	583	552	558	557	557
	9	1567	923	783	819	810	810	810
	10	85	300	333	340	342	343	343
	11	87	312	300	289	290	290	290
	12	262	466	583	552	558	557	557
	13	40	82	63	70	69	69	69
	14	40	46	46	50	51	52	52
	15	754	529	421	456	448	448	448
	16	812	394	362	362	362	362	362
	17	103	306	305	298	296	295	295
	18	738	534	417	448	442	443	443
	19	897	694	695	702	704	705	705
**Convergence** **index**		Inf	0.06	0.02	0.01	0.00	0.00	0.00

Based on the convergence index mentioned in Eq (65), it can be observed from Fig 4 that the algorithm terminates at iteration 18, indicating that this solution algorithm can solve the proposed traffic assignment problem efficiently and can be easily applied to other networks. And the value-related change process of convergence index and link flow is shown in Table 2.

The equilibrium route flow distributions for GVs and EVs are represented in Table 3. Moreover, the effect of travelers’ risk attitude toward network uncertainty on route choice behavior is also studied. As Table 3 shows, there is some difference in the route choice preference among these three types of travelers, especially for the EV drivers. For example, between OD pair (1,2), more EV drivers with LR use the route 2 while more with MR and HR use the route 1. Due to the less initial SOC for LR EV drivers, the initial energy cannot meet the requirement to reach the destination without charging, thus it needs to choose a route that covers charging stations. It should be noted that since the usable route sets for three types of EV drivers are different from each other, the mean and standard deviation for generalized route travel cost are not listed herein for lack of space. As for GV drivers, between OD pair (1,3), more GV drivers with LR use the route 2 (a low mean but high standard deviation) while more with MR and HR use the route 1 (a high mean but low standard deviation), indicating that the higher degree of risk-aversion of a traveler is, the more reluctant to choose the route with high standard deviation. Consistent with results in the previous studies (e.g., Lo et al. [5]), some routes with high standard deviation are still used by the traveler whose degree of risk-aversion is higher, it is reasonable in the sense that both types of routes achieve the same within budget cost reliability and require the same travel cost budget.

**Table 3:** Equilibrium route flows under entire network capacity degradation.

OD pairs	Route	GVs					EVs		
		Flow #LR	Flow # MR	Flow #HR	Generalized cost mean	Generalized cost st. dev.	Flow #LR	Flow # MR	Flow #HR
**(1,2)**	1	151	118	39	40.71	0.92	0	27	9
	2	5	3	1	44.07	1.45	13	1	0
	3	1	0	0	46.15	1.40	11	1	0
	4	0	0	0	50.45	0.56	6	0	0
	5	0	0	0	51.13	0.43	10	0	0
	6	1	1	0	45.82	1.34	0	1	0
	7	0	0	0	47.91	1.29	0	0	0
	8	0	0	0	52.17	0.09	0	0	0
**(1,3)**	1	136	127	50	45.35	0.83	23	35	8
	2	152	94	23	45.22	1.41	25	15	3
	3	2	2	1	49.52	0.57	20	0	2
	4	1	1	1	50.19	0.44	11	0	2
	5	26	18	5	46.97	1.30	0	11	2
	6	0	1	0	51.24	0.14	0	0	3
**(4,2)**	1	36	60	36	47.44	0.05	0	36	12
	2	179	108	21	45.81	1.41	20	7	1
	3	22	14	3	47.89	1.36	16	3	1
	4	0	0	0	52.18	0.45	9	0	1
	5	0	0	0	52.85	0.26	16	0	1
**(4,3)**	1	78	59	19	41.69	0.71	0	12	3
	2	1	1	0	46.51	0.12	0	3	1
	3	0	0	0	47.08	0.76	5	1	0
	4	1	0	0	46.95	1.37	3	0	0
	5	0	0	0	51.24	0.46	5	0	0
	6	0	0	0	51.91	0.28	8	0	0

Note: Since the usable route sets for three types of EV drivers are different from each other, the mean and standard deviation for generalized route travel cost are not listed herein for lack of space.

Additionally, in order to investigate the impact of local network capacity degradation on travelers’ route choice, it is assumed that maximum degradable coefficient *θ*_*a*_ = 0.45 for link 7 and the other link capacities have not been degraded. The other parameters are the same as those that are used above. As a result, route 5 between OD pairs (1,2), route 4 between OD pairs (1,3), routes 1 and 5 between OD pairs (4,2), and routes 2 and 6 between OD pairs (4,3) are directly influenced by the link degradation. The generalized cost standard deviations of these routes are obviously higher than which of other routes. As shown in Table 4, the route with minimum generalized cost is most popular for GV drivers when the standard deviation of generalized cost equals to zero. When the route has a lower mean compared with a route with high mean and 0-standard deviation, it still can be chosen by GV drivers although its standard deviation is over zero, such as routes 5 and 8 between OD pairs (1,2), and the choice difference narrows with the increase of travelers’ risk-aversion degrees. Moreover, the route with high mean and high standard deviation is least attractive among the route set, such as route 5 between OD pairs (4,2) and route 6 between OD pairs (4,3).Similarly, compared with EV drivers with MR and HR, the EV drivers with LR have the charging demand, hence their route choice is distributed among the routes with at least one charging station, and shows different characteristics. It is important to note that the ratios of scaling parameters between top level and bottom level for different types of travelers lie within the 0±1 range (respectively are 0.743 (GV drivers with LR), 0.744 (GV drivers with MR), 0.744 (GV drivers with HR), 0.814 (EV drivers with LR), 0.745 (EV drivers with MR), 0.746 (EV drivers with HR)), meeting the condition for consistency with utility maximization in the NL model, and indicating that it is appropriate to adopt the nested modelling structure to analyze travelers’ route choice when relatively larger perceived error difference among route alternatives exists.

**Table 4:** Equilibrium route flows under local network capacity degradation.

OD pairs	Route	GVs					EVs		
		Flow #LR	Flow # MR	Flow #HR	Generalized cost mean	Generalized cost st. dev.	Flow #LR	Flow # MR	Flow #HR
**(1,2)**	1	47	36	12	38.34	0.00	0	7	2
	2	35	27	9	40.61	0.00	10	7	2
	3	17	13	4	45.69	0.00	10	4	1
	4	9	7	2	50.62	0.00	9	0	1
	5	8	6	2	51.24	0.22	10	0	1
	6	24	19	6	43.13	0.00	0	5	1
	7	12	9	3	48.22	0.00	0	3	1
	8	6	5	2	53.15	0.00	0	0	1
**(1,3)**	1	99	76	25	42.52	0.00	20	27	5
	2	72	55	18	44.84	0.00	21	17	3
	3	36	28	9	49.77	0.00	20	2	2
	4	33	25	8	50.39	0.22	18	2	2
	5	51	39	13	47.36	0.00	0	13	3
	6	26	20	7	52.29	0.00	0	1	2
**(4,2)**	1	45	34	11	48.11	0.22	0	12	3
	2	101	78	26	42.90	0.00	16	19	5
	3	50	39	13	47.99	0.00	15	11	3
	4	25	19	6	52.92	0.00	14	2	2
	5	16	12	4	53.54	0.22	15	0	1
**(4,3)**	1	33	25	8	39.38	0.00	0	6	2
	2	10	8	2	47.25	0.22	0	3	1
	3	15	12	4	44.81	0.00	5	3	1
	4	11	9	3	47.13	0.00	4	2	1
	5	6	4	1	52.06	0.00	5	0	0
	6	4	3	1	52.68	0.22	5	0	0

Note: Since the usable route sets for three types of EV drivers are different from each other, the mean and standard deviation for generalized route travel cost are not listed herein for lack of space.

**Fig 5:**
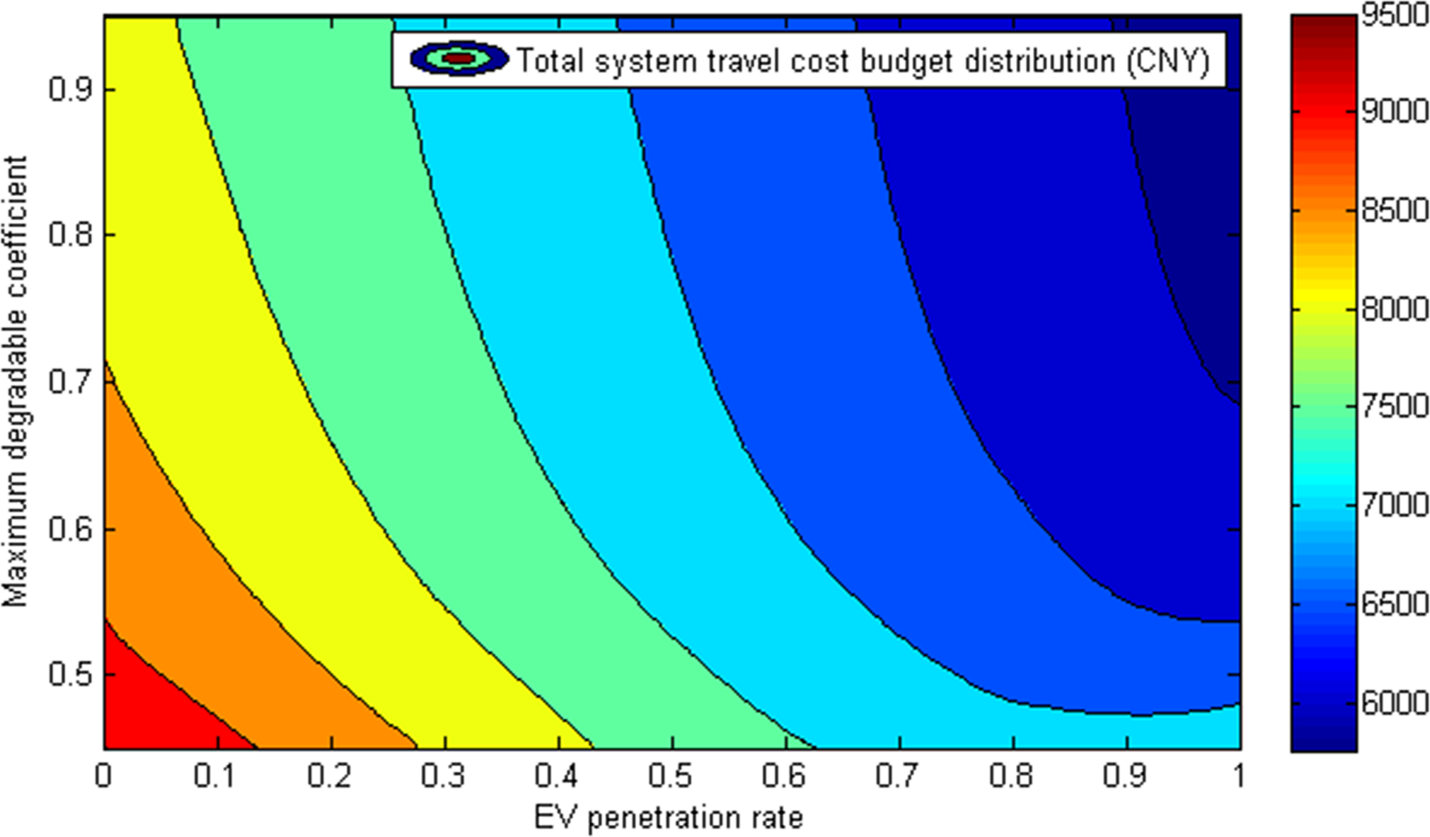
Total system travel cost budget variation with EV penetration rate and maximum degradable coefficient.

Fig 5 summarizes the effects of EV penetration rate and maximum degradable coefficient for the transportation network on total system travel cost budget (i.e., the amount of routes’ travel cost budgets for all users). It can be concluded that the higher the EV penetration rate is, the lower the total system travel cost budget achieves, so is the maximum degradable coefficient. It is particularly worth mentioning here that the lowest total system travel cost budget is not at the highest maximum degradable coefficient or the maximum EV penetration rate unilaterally. Instead, the total system travel cost budget achieves lowest when there is a higher EV penetration rate and network reliability simultaneously. The reason behind this phenomenon might be that when the transportation network is more reliable, the variation related to the generalized route travel cost will be reduced. And when more EVs are introduced, the travel cost related to energy consumption will decrease.

**Fig 6:**
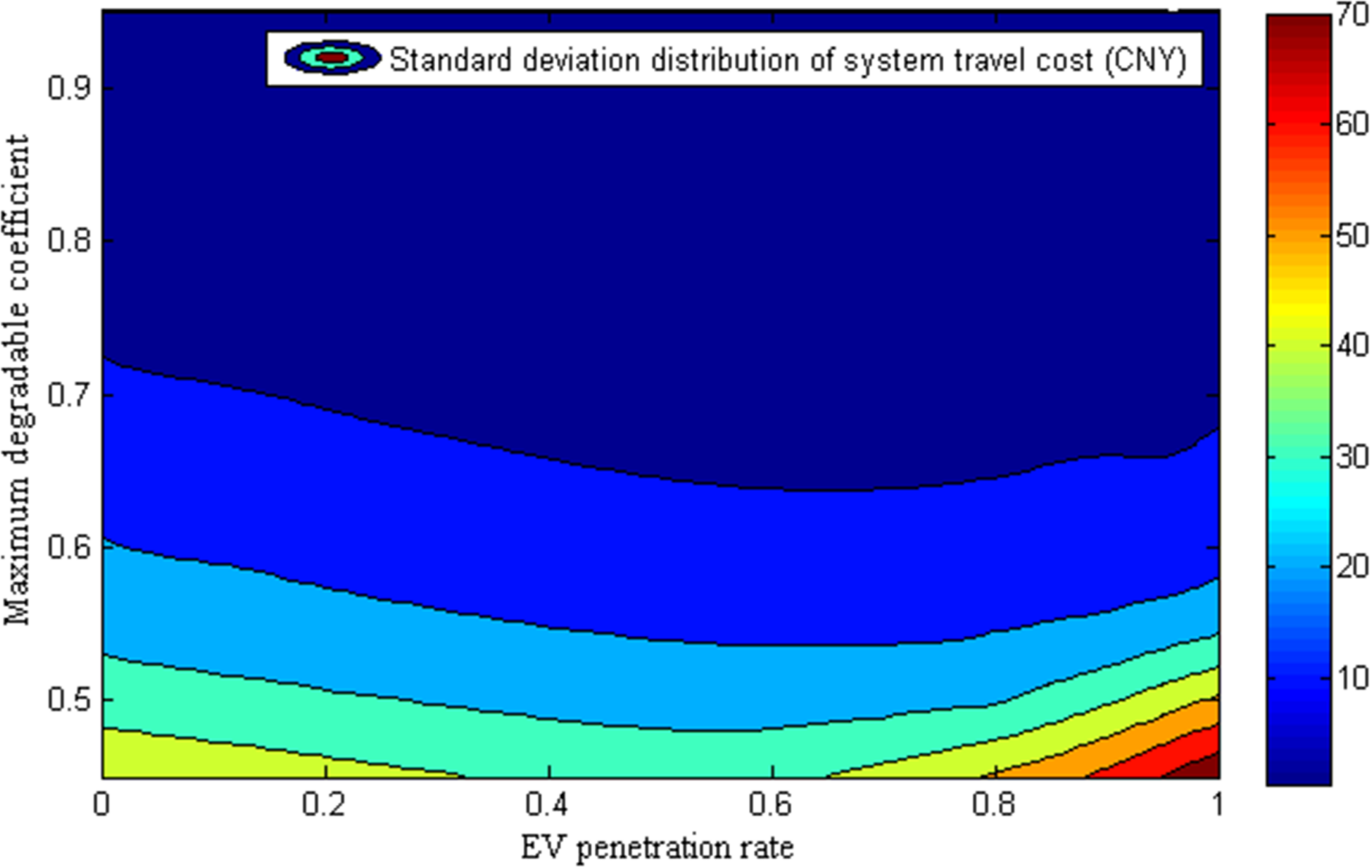
Standard deviation variation of system travel cost with EV penetration rate and maximum degradable coefficient.

Fig 6 displays the standard deviation distribution of system travel cost under various EV penetration rates and maximum degradable coefficients. Different from the variation pattern of total system travel cost budget in Fig 5, the lower EV penetration rate and higher maximum network degradable coefficient lead to the reduction for total system travel cost standard deviation. Due to the usable routes for EV drivers change with the network conditions (EV penetration rate and maximum degradable coefficient), the variation trend is in the form of highly nonlinear nature. Moreover, when the reliability of transportation network is higher, the influence of EV penetration rate on system standard deviation is not obvious.

Therefore, from what has been discussed above, it would be reasonable to believe that the widespread adoption of EVs can cut down the total system travel cost effectively only when the transportation network is more reliable.

**Fig 7:**
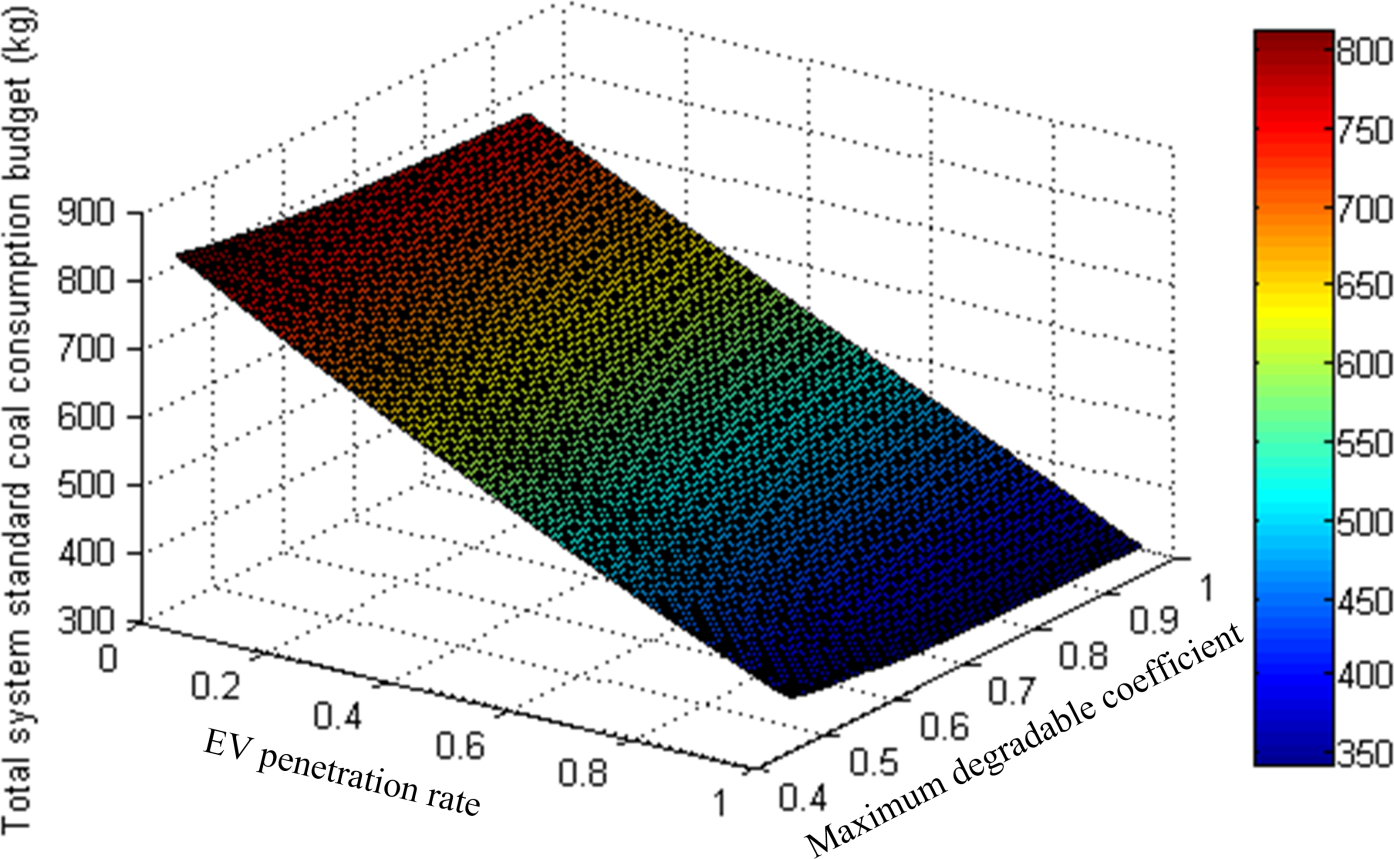
Total system standard coal consumption variation with EV penetration rate and maximum degradable coefficient.

Furthermore, given that the energy prices for gasoline and electricity might differ greatly in different countries or regions, both the gasoline consumption of GVs and electricity consumption of EVs are converted into coal equivalent consumption to verify the impacts of EV penetration rate and maximum degradable coefficient on total system energy consumption [28]. Particularly, the conversion formulas for GVs and EVs are adopted as $\gamma_{a}^{G}=1.471\times e_{a}^{G}$ and $\gamma_{a}^{E}=0.326\times e_{a}^{E}/\left[(1-6.62\%)\times 97\%\right]$, respectively. The former formula means that 1.471kg standard coal will be consumed to produce 1kg gasoline, and the latter formula indicates that the energy generated by 0.326kg standard coal equals to which by 1kwh electricity. Meanwhile, 6.62% is the power line loss and 97% is the charge-discharge efficiency of EV battery. Consequently, the total system standard coal consumption variation with EV penetration rate and maximum degradable coefficient is exhibited in Fig 7. As shown, the total system standard coal consumption is most affected by EV penetration rate compared to maximum degradable coefficient. However, both the increases of EV penetration rate and maximum degradable coefficient will decrease the total system standard coal consumption, indicating that the popularization of EVs in large scale can save total system energy consumption significantly. By contrast, since the increase of EV penetration rate can save more total system energy consumption (the energy efficiency of EVs is higher than GVs), the effect of improving the reliability of transportation network is not remarkable although the total system energy consumption can be reduced a little.

**Fig 8:**
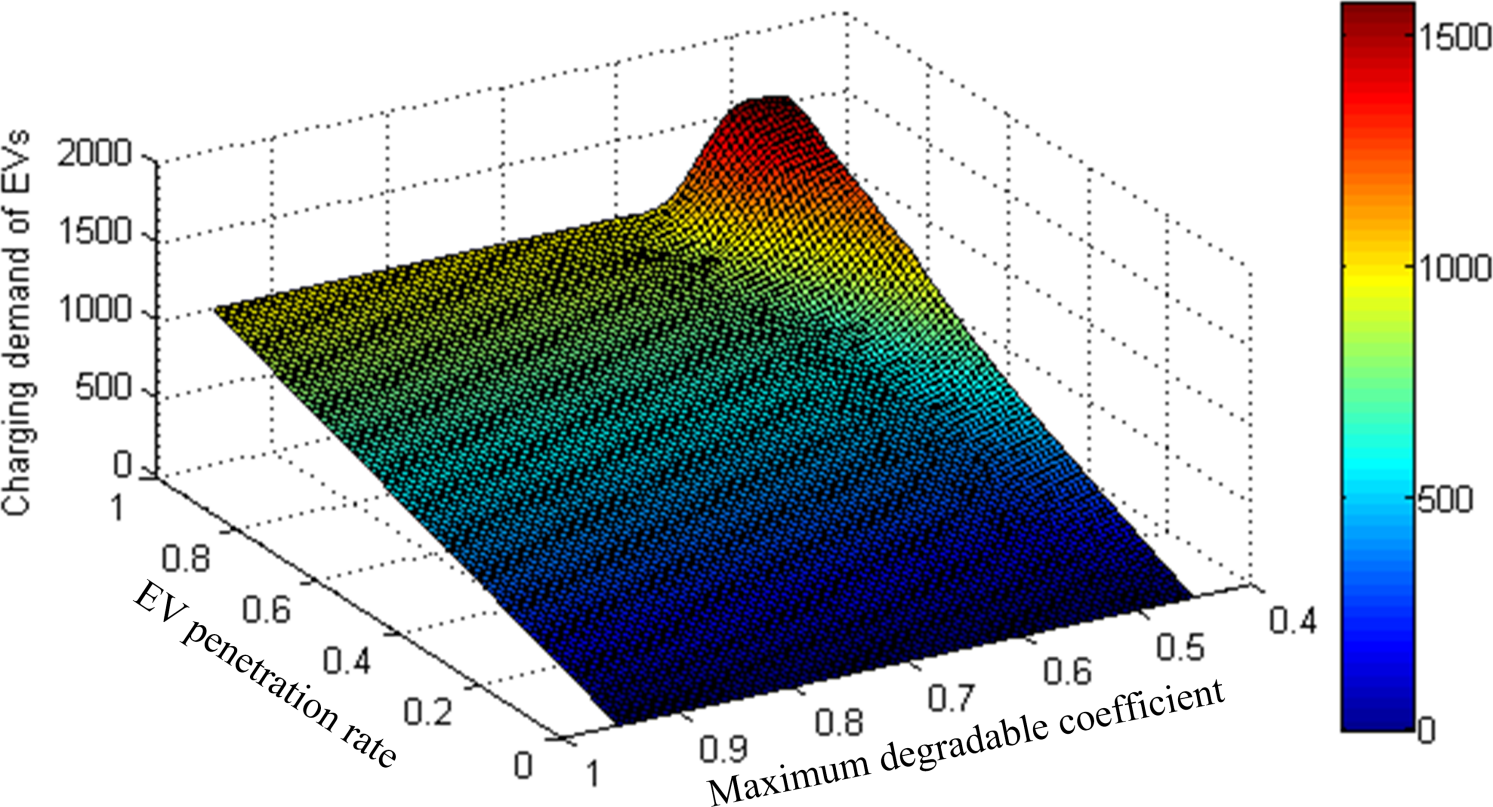
EV charging demand with EV penetration rate and maximum degradable coefficient.

Fig 8 shows the EV charging demand variation with different network conditions (EV penetration rate and maximum degradable coefficient). It can be seen that maximum degradable coefficient has no significant impact on EV charging demand when EV penetration rate is at a lower level. However, with the increase of EV penetration rate, the poor network operation condition will result in the increase of EV charging demand. Therefore, to provide a better charging service to the majority of the network users, it is necessary to consider both the influences of EV penetration rate and maximum degradable coefficient on EV charging demand when locating EV charging infrastructures. Specifically, EV charging demand needs to be estimated under different combinations of EV penetration rate and maximum degradable coefficient to test the serviceability of EV charging infrastructures.

## Conclusions

The introduction of EVs calls for fundamental changes to the existing traffic assignment modeling tools for accommodating urban mixed gasoline and electric vehicular flows, especially in the degradable transportation network. This paper has begun to address this issue by presenting a reliability-based network equilibrium framework for capturing travelers’ route choice behaviors and the induced constrains with the addition of EVs. Specifically, given the unique energy consumption characteristics of EVs, the flow-dependent energy consumption cost and travel time, charging demand and charging time are given fully consideration in generalized route travel cost calculation in degradable network, while only the former two are incorporated in for GVs. The means and variances of link and route travel time are derived firstly, and the distributions of link and route energy consumption, as well as the EV charging time distribution are presented subsequently. Furthermore, based on a nested route choice structure, the route travel cost budgets are viewed as the principle of travelers’ route choice, and a reliability-based traffic assignment model with the addition of EVs is formulated. Finally, through developing a heuristic solution algorithm from the MSWA to solve the proposed model, the effects of travelers’ risk attitude, transportation network reliability and EV penetration rate on network performance are fully analyzed based on the example application. In the numerical example, the ratios of scaling parameters between top level and bottom level for different types of travelers lie within the 0±1 range in NL model, demonstrating that it is suitable to employ the nested modelling structure to analyze travelers’ route choice for accommodating different uncertainty degrees of the routes with and without degradable road links. And the equilibrium route flow distributions under different network degradation conditions reveal that the initial SOC and energy demand will determine the usable route set for an EV driver to reach the destination and further reshape the network traffic flow. In this study, a route with high mean but low standard deviation is more popular among the GV drivers with higher degree of risk-aversion. But some routes with high standard deviation are still used by the traveler whose degree of risk-aversion is higher due to the same within budget cost reliability and the same travel cost budget. Besides, the study on impacts of EV penetration rate and maximum degradable coefficient on total system travel cost budget and system travel cost standard deviation shows the highly nonlinear nature of these influencing factors, indicating that the widespread adoption of EVs can cut down the total system travel cost effectively when the transportation network is more reliable. Specifically, the total system travel cost is relatively low (nearly lower than 6000 CNY) when the network maximum degradable coefficient is higher than 0.9 and the EV penetration rate exceeds 70%. Meanwhile, the analysis of EV charging demand variation under different network conditions points out that it is necessary to take EV penetration rate and network reliability into consideration when planning EV charging infrastructures. In particularly, when the network maximum degradable coefficient is lower than 0.7 and the EV penetration rate exceeds 45%, there will be an obvious increase of EV charging demand.

The presented modelling method in this paper is helpful to understand travelers’ route choice behavior in unreliable transportation network with the addition of EVs, and further aids in the travel demand forecasting and transportation development plan evaluation.
